# A Fully-Coupled Electro-Mechanical Whole-Heart Computational Model: Influence of Cardiac Contraction on the ECG

**DOI:** 10.3389/fphys.2021.778872

**Published:** 2021-12-16

**Authors:** Robin Moss, Eike Moritz Wülfers, Steffen Schuler, Axel Loewe, Gunnar Seemann

**Affiliations:** ^1^Institute for Experimental Cardiovascular Medicine, University Heart Center Freiburg - Bad Krozingen, Medical Center–University of Freiburg, Freiburg, Germany; ^2^Faculty of Medicine, University of Freiburg, Freiburg, Germany; ^3^Institute of Biomedical Engineering, Karlsruhe Institute of Technology (KIT), Karlsruhe, Germany

**Keywords:** cardiac mechanics, cardiac electrophysiology, electrocardiography, computational whole-heart modeling, electro-mechanical coupling, biomedical engineering

## Abstract

The ECG is one of the most commonly used non-invasive tools to gain insights into the electrical functioning of the heart. It has been crucial as a foundation in the creation and validation of *in silico* models describing the underlying electrophysiological processes. However, so far, the contraction of the heart and its influences on the ECG have mainly been overlooked in *in silico* models. As the heart contracts and moves, so do the electrical sources within the heart responsible for the signal on the body surface, thus potentially altering the ECG. To illuminate these aspects, we developed a human 4-chamber electro-mechanically coupled whole heart *in silico* model and embedded it within a torso model. Our model faithfully reproduces measured 12-lead ECG traces, circulatory characteristics, as well as physiological ventricular rotation and atrioventricular valve plane displacement. We compare our dynamic model to three non-deforming ones in terms of standard clinically used ECG leads (Einthoven and Wilson) and body surface potential maps (BSPM). The non-deforming models consider the heart at its ventricular end-diastatic, end-diastolic and end-systolic states. The standard leads show negligible differences during P-Wave and QRS-Complex, yet during T-Wave the leads closest to the heart show prominent differences in amplitude. When looking at the BSPM, there are no notable differences during the P-Wave, but effects of cardiac motion can be observed already during the QRS-Complex, increasing further during the T-Wave. We conclude that for the modeling of activation (P-Wave/QRS-Complex), the associated effort of simulating a complete electro-mechanical approach is not worth the computational cost. But when looking at ventricular repolarization (T-Wave) in standard leads as well as BSPM, there are areas where the signal can be influenced by cardiac motion of the heart to an extent that should not be ignored.

## 1. Introduction

The ECG is one of the most frequently used tools in medicine to assess electrical function and dysfunction of the heart. As cardiomyocytes sequentially depolarize electrically prior to contraction, they generate electric fields, leading to transient voltage gradients. The consecutive activation of atria and ventricles is represented by the P-Wave and QRS-Complex in the ECG, respectively. After some time, the cardiomyocytes repolarize to their resting state, once again leading to transient voltage gradients, which for ventricular repolarization is visible in the ECG as the T-Wave.

But as the heart deforms during contraction, the sources of the electrical field will move relative to the body surface. Such movements may cause a change in the influence of individual areas of the heart on body surface potentials between depolarization and repolarization. This effect would be expected to materialize in particular during the T-Wave, as it aligns with peak contraction. To better understand the processes occurring during a heart beat, computational modeling can be used. Yet, within existing models a static end-diastatic representation of the heart is normally used to represent the underlying cardiac anatomy (Keller et al., 2010, 2012; Maffessanti et al., 2013; Andlauer et al., 2018; Gillette et al., 2021).

Several studies have tried to estimate the impact of cardiac displacement on the ECG, coming to different conclusions. Using simple models and calculating a so called pseudo-ECG, only minor effects of heart movement were observed by Oliveira et al. (2013) and Favino et al. (2016). Similarly, minor alterations were observed in a two-dimensional model by Smith et al. (2003). In contrast, creating a more advanced three-dimensional model, based on MRI data, Wei et al. (2006) saw distinct effects of deformation on the Wilson leads close to the heart, especially during ST-Segment and T-Wave. A similar approach, presented by Keller et al. (2011), also extracted ventricular deformation data from MRI and mapped those onto a model of the ventricles. But, in contrast, they predominantly reported changes in the T-Wave in the Einthoven II lead.

In recent years, cardiac mechanical modeling has steadily advanced in terms of complexity (Gurev et al., 2015; Augustin et al., 2016; Gerach et al., 2021) and physiological accuracy (Fritz et al., 2014; Pfaller et al., 2019). The present study builds on this, and presents a human electro-mechanically coupled 4-chamber whole-heart model, embedded into the torso. We aim to overcome shortcomings of previous studies by using an accurate three-dimensional representation of the heart and torso, a higher geometrical mesh resolution, better verification, and by accurately representing deformation and with it the movement of the field origins responsible for the ECG signals on the torso surface. To do so, we simulate on a geometrical model from a healthy volunteer and validate against ECG recordings from the same volunteer. Resulting contractile motion is further validated against literature values in terms of circulation, atrioventricular plane displacement (AVPD), and twisting motion.

**Figure 1:**
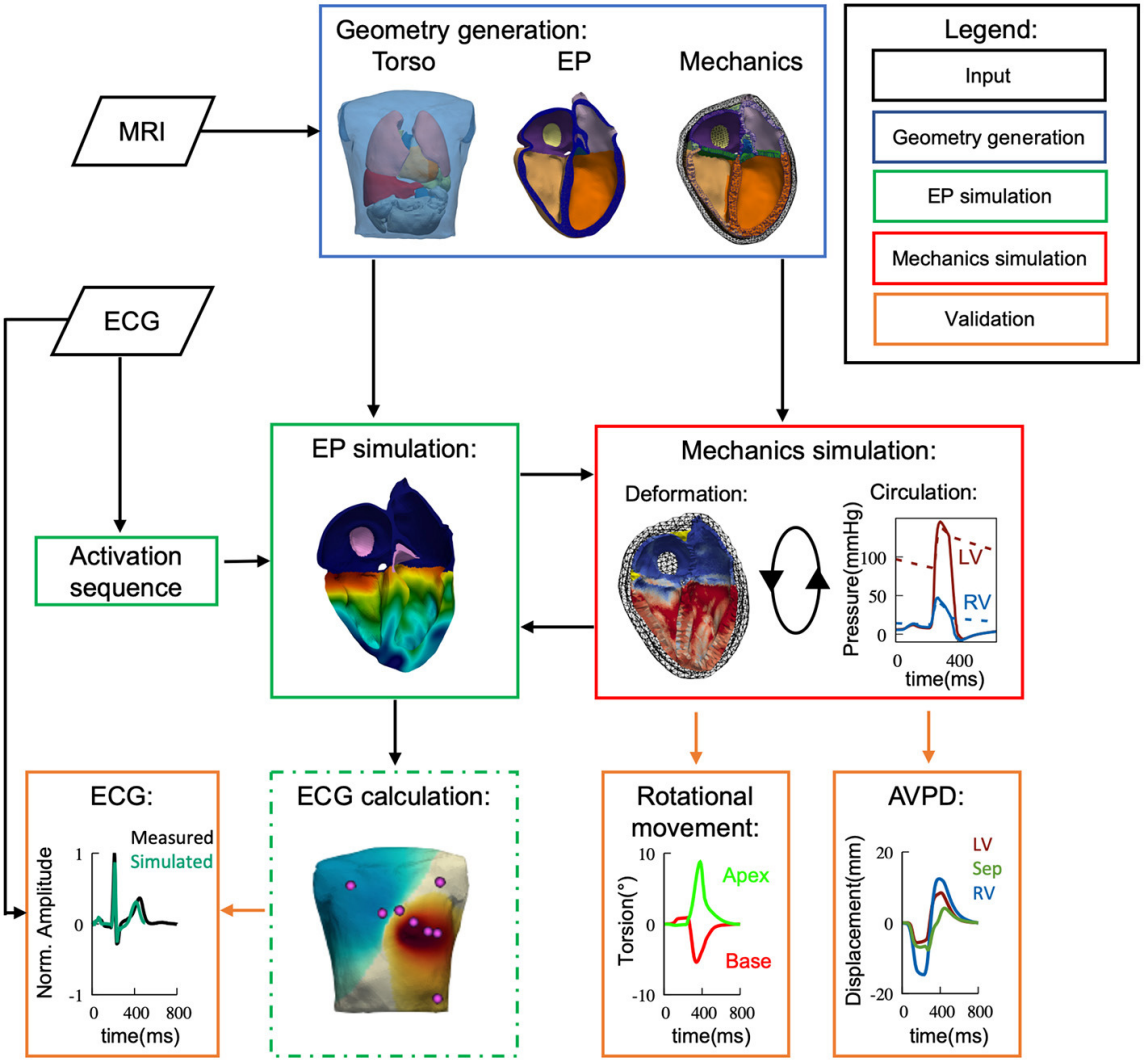
Flow diagram depicting the used methods of simulation and validation within this study. Based on measured data (black) we have developed a 4-chamber fully coupled electro-mechanical (green/red) computational model, embedded within the torso. MRI and ECG were recorded from the same volunteer. The electrophysiological side of the model was then validated against the ECG data and the mechanical side against literature values in terms of cardiac output, valve plane movement and ventricular rotational movement.

By doing so, we intend to offer a better understanding of the impact of cardiac contraction in terms of clinically used ECG leads and the body surface potential map (BSPM), as the observations of previous studies were inconclusive and partially contradicting. A schematic diagram of the modeling and validation workflow can be seen in Figure 1. Further, as computational modeling has been proposed more and more as a tool to help with diagnostics in clinics, the scientific field is advancing closer to the goal of making personalized modeling openly available (Dössel et al., 2021). The development of a fully electro-mechanically coupled *in silico* model, instead of extracting contractile motion from image data, generates a platform for such personalized investigations, as the ECG presents itself as an ideal point of translational interaction between the digital and real world. But, as the mechanical side of this fully coupled approach is still computationally heavy compared to the electrophysiological one, its necessity needs to be evaluated. Thus, to quantify the potentially overlooked influence that deformation has on the resulting BSPM, we compare our approach of using a dynamic heart model with three non-deforming states of the heart cycle (end-diastatic: before atrial contraction, end-diastolic: after atrial contraction, and end-systolic: after ventricular contraction). We chose these three states as they coincide with the three major sections of the ECG, i.e., P-Wave, QRS-Complex and T-Wave. The results of this comparison may aid model selection for future computational studies investigating electrophysiological function as well as dysfunction, which overlap with different contractile states of the heart.

## 2. Methods

### 2.1. Volumetric Models

**Figure 2:**
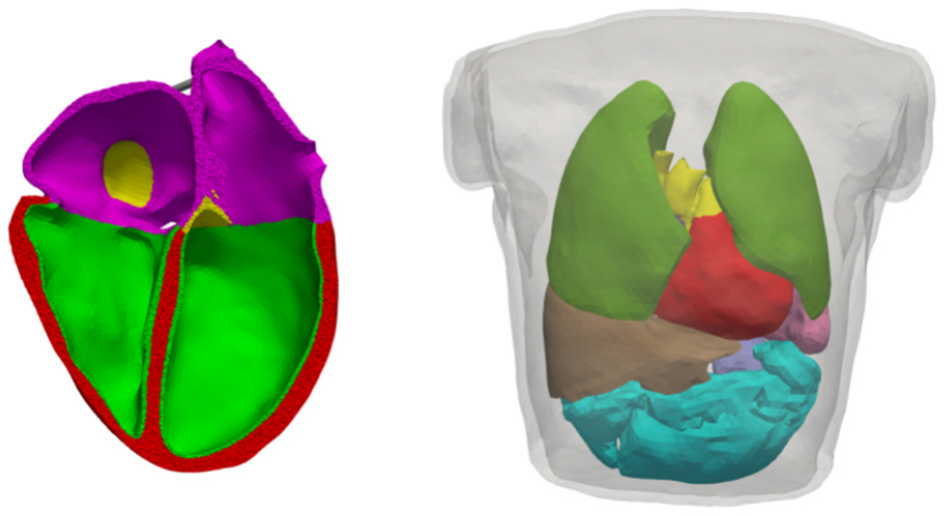
Volumetric geometries used for electrophysiological simulations. Left: Cut of the heart mesh; myocardium where the *endo* variant of the OVVR model is used (green), myocardium where the *epi* variant of the OVVR model is used (red), atrial wall (purple), blood vessels (yellow). Right: Torso mesh as used for forward calculation of the ECG; lungs (dark-green), liver (brown), spleen (lavender), intestine (turquoise), others as left side. Both models are based on the same volunteer as published by Keller et al. (2012).

**Figure 3:**
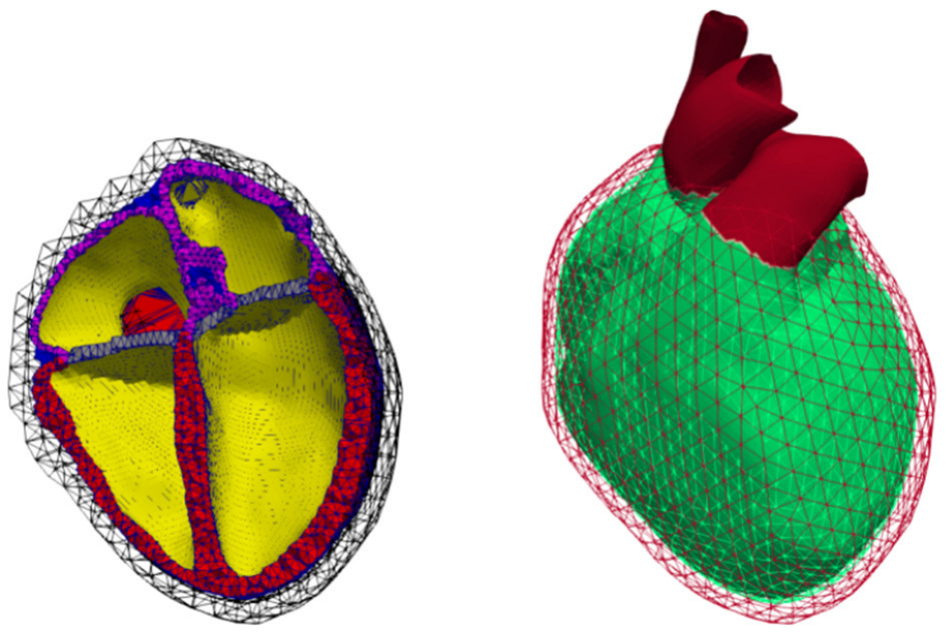
Volumetric geometry used for continuum mechanics. Left: Cut open mesh used to calculate deformation; cavities (yellow), ventricular walls (red), atrial walls (purple), fat (green), valves (blue), non-cardiac tissue (black mesh). Right: Representation of the Dirichlet boundary condition marked in red comprising the outer layer of the surrounding non-cardiac tissue as well as all blood vessels protruding that layer.

The geometrical models, used for electrophysiology simulations (i.e., electrical excitation and propagation; Figure 2), as well as for mechanics (Figure 3), are based on data from the same volunteer (Keller et al., 2011, 2012). Since the heart model as used by Keller et al. (2011) was primarily generated for electrophysiological simulations, it could not be used for mechanics due to its lack of aorta and pulmonary artery, which needed to be re-segmented. This was done by re-segmenting parts of the same set of MRI data using *Seg3D* v2.4.4 (CIBC, 2016). *Blender* (Blender Online Community, 2020) was then used to smooth the geometrical surfaces as well as repair segmentation errors. Finally, *Gmsh* (Geuzaine and Remacle, 2009) was used to create a tetrahedral mesh of the heart. The corresponding torso model as published by Keller et al. (2012) could mostly be reused, with the alteration of adding a thin layer of elements surrounding the heart, which is used as to not re-mesh the complete torso for each time step during deformation.

In total, the mesh of the heart used for electrophysiological simulations (Figure 2) comprised 1.5M points and 8.4M elements, with an average edge length of 0.7 ± 0.1 mm (average ± standard deviation). The torso accounted for another 900k tetrahedra with an average edge length of 7.93 ± 3.15 mm.

The mechanical part of the simulation is computationally more demanding, hence a coarser mesh was generated by retopologizing the high resolution heart surface from the electrophysiology model using *Instant Meshes* (Jakob et al., 2015). The resulting mechanical mesh comprising 121k elements was created in the same way as the electrical one. Out of these, 56k elements are part of the ventricles, 21k of the atria, and the rest form the aorta, pulmonary artery, veins, fat, and surrounding non-cardiac tissue, see Figure 3. As the elements in the later described model of continuum mechanics are quadratic tetrahedrons, this results in 210k nodes and 525k degrees of freedom (DOF) in total, with the DOF not including nodes for which Dirichlet boundary conditions were imposed. Dirichlet boundary conditions were applied for all nodes on the outside surface of the non-cardiac tissue and all nodes of vessels protruding from the aforementioned surface (cf. red surface in Figure 3, right). For both heart models, the ventricular myocyte orientation was defined based on Streeter et al. (1969) as was done by Fritz et al. (2014); Pfaller et al. (2019), i.e., assuming a gradual transmural twist from 60° (endocardium) to −60° (epicardium).

For the atrial main myocyte orientation (f), the rule-based algorithm published by Wachter et al. (2015) was employed. The additionally needed sheet-normal (sn) myocyte orientation was defined to be in same direction as a gradient calculated using a Laplace equation between the epicardial and endocardial surface. The orthonormal coordinate system was then completed by calculating the myocardial sheet (s) orientation as the cross product of the main (f) and sheet-normal (sn) myocyte orientation vectors.

### 2.2. Electrophysiology and Active Tension Modeling

#### 2.2.1. Cardiac Tissue Electrophysiology

Electrical excitation and wave propagation in the myocardium were simulated as part of our electro-mechanical simulation framework (Gerach et al., 2021). The electrophysiological part is based on the verified framework *acCELLerate* (Seemann et al., 2010; Niederer et al., 2011). Tissue parameters were set such that conduction velocities (CV) of 800 mm/s along myocyte orientation and 550 mm/s in transverse directions were reached. Further information of the underlying monodomain formulation and numerical details are included in the Supplementary Materials (section 1.1.1).

For atrial myocytes, the cell model by Courtemanche et al. (1998) (CRN) was used to calculate the membrane currents, with alterations as described in van Oosterom and Jacquemet (2009), as the model would not be able to reach limit cycle (“steady state”) otherwise. Ventricular myocyte membrane currents were calculated using the O'Hara–Virág–Varró–Rudy (OVVR) model (O'Hara et al., 2011), with alterations to the sodium current as published by Dutta et al. (2017). The alterations were necessary to achieve physiological CV in tissue at the mesh resolution used in our model. Since the OVVR model has an *epi* and an *endo* variant of myocyte properties, the ventricular walls were subdivided into a layer where the *endo* variant (accounting for 20 % of the wall thickness), and a layer where the *epi* variant was assigned (remaining; cf. Figure 2). Further, an apico-basal heterogeneity in the slow delayed rectifying potassium channel conductance *g*_Ks_ was introduced, causing a shortening of action potential duration (APD) toward the apex by approximately 17%. An apico-basal APD gradient (with strong contribution of *I*_Ks_) was observed by Szentadrassy et al. (2005) and shown by Keller et al. (2011) to result in physiological T-Wave morphology in the simulated ECG. Before initiating whole-heart simulations, all cell models were paced in a single cell environment until limit cycle was reached.

Excitation in the whole-heart simulations was initiated using a transmembrane stimulus current in the sinus node area of the atria and, then after assuming an atrio-ventricular node delay of 120 ms, the ventricles were stimulated using a pattern mimicking the activation via Purkinje–muscular junctions. This activation pattern was calculated using the optimization scheme published by Kahlmann et al. (2017), with the recorded ECG signals from Keller et al. (2012), i.e., from the same volunteer the geometry was based on, as a target. Here, the node density, maximal tree height, CV, and activation time of an activation sequence, mimicking the Purkinje network, are iteratively optimized. In each iteration, ventricular local activation times are estimated based on the current Purkinje tree using a fast marching algorithm. Combining these activation times with predefined action potential snippets yields an estimate of *V*_m_ distribution, from which an ECG is calculated using a lead-field approach. This ECG estimate is compared with the target ECG.

Based on the measured heart rate in the ECG, the stimulus cycle length was set to 850 ms, resulting in a heart rate of approximately 70 bpm. The instances of the cell models that describe the currents at each node of the geometry were initialized in an isolated and static mode. Thus, the impact of *strong coupling* was not present during initialization. Therefore, the electrophysiological side of the fully-coupled model took another 3 beats until it stabilized, determined by comparing the resulting ECG of two consecutive heart cycles (root mean square error < 0.1 mV), after being coupled to the mechanics model.

#### 2.2.2. Forward Calculation of ECG

The forward problem of the ECG (i.e., the calculation of body surface potential maps (BSPM) from transmembrane voltages computed with the monodomain equation) was solved as published by Keller et al. (2012) with few minor alterations: Since excitation propagation was already calculated on a tetrahedral mesh, no further interpolation between the meshes of different types was needed. The deformed mesh of the heart was inserted into the torso mesh by remeshing the non-cardiac tissue layer surrounding the heart as described in section 2.1.

ECG leads were derived from the resulting BSPM as traces of the extracellular potentials over time at fixed locations on the body surface. Wilson's central terminal was used as reference potential for BSPM voltages. Numerical details on the solution of the forward problem can be found in the Supplementary Materials (section 1.1.2).

#### 2.2.3. Tension Generation

In order to calculate the force generated by the myocytes during contraction, the tension model by Rice et al. (2008) was coupled to the electrophysiological models. For all models, the temperature was set to a physiological value of 37°C. As the tension model itself has a stretch and stretch/shortening-velocity dependency, it was coupled to the mechanical part of the simulation, which is described later. Therefore, myocyte length changes and their derivatives (i.e., velocity) were interpolated using quadratic shape functions from the mechanical to the electrophysiological mesh for each time step (Gerach et al., 2021).

Parameters of the ventricular tension model were set as published by Timmermann et al. (2017) under consideration of the formulation for *strong coupling*. This means, that, additional to the previous stated coupling, the length and velocity dependent modulation of troponin C binding was calculated within the tension model, replacing the original formulation in the electrophysiological model. As the original formulation for troponin C binding in the electrophysiological model is neither length nor velocity dependent, this can lead to an alteration of the resulting AP, depending on contraction. From a physiological point of view, *strong coupling* is a more accurate representation of the underlying mechanisms. But as calcium dynamics in electrophysiological models are often quite delicate, such modification can disturb their balance and result in unphysiological behavior.

In terms of the atrial tension model, the original rat species parameters (Rice et al., 2008) were applied, as proposed by Brocklehurst et al. (2015). Yet, *strong coupling* led to instabilities in the underlying electrophysiological model (CRN), manifesting as the model being unresponsive to pacing after 2 fully coupled beats. Therefore, *strong coupling* was not applied to atrial cells.

### 2.3. Mechanics

The underlying parameters which differentiated from the used electro-mechanical framework and its major components as presented by Gerach et al. (2021) are described in the following. The mechanical part of the framework was based on the verified mechanical framework published by Fritz et al. (2014); Land et al. (2015). In short, given Equation (7) from Fritz et al. (2014),


(1) $$\begin{array}{r} M\ddot{u}+f^{\text{intern}}-f^{\text{extern}}=0, \end{array}$$

where the major components which have to be defined to calculate the deformation (***u***) are the geometry (***M***; being the mass matrix calculated based on the spatially discretized geometry shown in section 2.1), the internal stress (***f***^intern^; describing the stress generated due to active contraction and passive properties of the tissue), and the external stress (***f***^extern^; accounting for all stresses due to blood pressure or interactions with surrounding non-cardiac tissue). A detailed investigation, based on a domain specific benchmark problem as posed by Land et al. (2015), regarding whether the resolution of the mesh is sufficient can be found in the supplement of Gerach et al. (2021). There, by benchmarking different resolutions, they come to the conclusion that their geometry is of high enough resolution. In comparison, the geometry used within this study has roughly 5 times the amount of quadratic elements and comprises 3.5 times the amount of DOF.

#### 2.3.1. Internal Stress

**Passive:** To describe its passive stress-strain relationship, the cardiac tissue is assumed to be nearly incompressible, anisotropic, and hyperelastic. There are numerous published material laws describing these properties, with the one by Usyk et al. (2000) being used for the ventricles as well as atria in this work, with the parameters being set according to Gurev et al. (2015). The choice for this specific material law was based on its behavior during our proposed initialization scheme. Other materials model would have needed to be re-parameterized, similar to as proposed by Kovacheva et al. (2020). For all other types of tissue, the energy function was characterized by using a neo-Hookean material (Gerach et al., 2021). See Supplementary Materials (section 1.2.1) for the respective energy functions and used parameters.

**Active:** The stress calculated due to active tension (*T*_k_) as described in 2.2.3 was linearly interpolated from the nodes of the electrophysiological mesh to the Gauss integration points of the quadratic elements of the mechanical mesh. For this, a constant mapping matrix between the two meshes was determined prior to any deformation. The tension was then added to the first element of the second Piola–Kirchhoff stress tensor (***S***), resulting in a contraction in main myocyte direction (see also Gerach et al., 2021).

#### 2.3.2. External Stress

**Surrounding Tissue:** As the heart is embedded in the pericardial sac, its influence on the motion of the heart has to be taken into account as well. As shown by Fritz et al. (2014) and Strocchi et al. (2020b) the inclusion of these influences is crucial, in order to be able to simulate a physiological contraction. Within our framework, this is done by modeling the interaction between the inner (i.e., heart-facing) surface of the non-cardiac tissue and the epicardial surface of the heart as a friction-less contact problem (Fritz et al., 2014; Pfaller et al., 2019; Gerach et al., 2021), yet different approaches, such as using spatially varying Robin boundary conditions, do exist (e.g., Strocchi et al., 2020b). For the friction-less contact problem, two surfaces based on the used geometry had to be defined: One being the heart-facing surface of the tissue surrounding the heart and the other being the epicardial surface of the heart (defined manually). As these surfaces are biologically equivalent to the pericardium and pleural surface, which form a lubricated laterally-movable contact surface, they will be hereinafter referred to as the pericardium. The gap function (***g***_N_) governs the acting force on the respective aforementioned surfaces in normal direction. The formulation as defined by Fritz et al. (2014) in Equation (24), was replaced with a non-linear continuous function, as it resulted in a better convergence for our used geometry. A more extensive description, as well as comparison between the old and new gap function can be found in the Supplementary Materials (section 1.2.2).

**Circulation:** To model effects of the circulatory system on cardiac mechanical load, a fully coupled closed-loop lumped model representing the blood flow through the heart chambers, the lungs, and the rest of the body was used as described by Gerach et al. (2021). Therefore, for our given geometry four distinct closed cavities were defined (inner surfaces of each of the four chambers of the heart), with the openings in the atria being closed via triangulation without adding additional nodes. All other distinct parts of the circulatory system are concentrated and represented by a series of diodes, resistors, and capacitances. The finite element mechanics model and the lumped circulation model are then coupled with the goal of iterative adaption of the respective cavity pressures, such that the in- and outflows match one another. A more detailed description can be found in the Supplementary Materials (section 1.2.3).

#### 2.3.3. Damping and Calculation of Deformation

In order to damp oscillations, the parameters of the Rayleigh damping matrix (α_R_***M***+β_R_***K***) as proposed by Fritz et al. (2014) in Equation (11) were adjusted. The coefficient α_R_ was set to 100 s^−1^ (Fritz et al., 2014; Gerach et al., 2021) and β_R_ to 0.04 s. It should be noted that ***K*** (i.e., the isotropic tangential stiffness matrix of the passive stresses) and the resulting damping are highly dependent on the state of contraction and the used material law, hence, making a direct comparison to previously published parameter values difficult. The values chosen for damping here were selected such that oscillation introduced by the external stresses were damped sufficiently. Especially during isovolumetric relaxation, the problem to solve becomes rather stiff. Here the interactions during the iterative coupling of the mechanics and circulation problems can manifest as oscillations which, without damping, would build up and result in diverging solutions. The subsequent deformation was then calculated using a Newmark-beta scheme as described in the supplement of Gerach et al. (2021), where the resulting non-linear system was solved using distributed supernodal LU decomposition (Li et al., 1999; Li and Demmel, 2003), provided in the *PETSc* software package (Abhyankar et al., 2018), as the direct solver such that the residual norm fulfilled ||***r***|| < 10^−8^.

The simulation of one heart beat took approximately 20 h using all cores on a second generation 64-Core AMD EPYC processor (7H12), varying depending on if the time step had to be reduced momentarily during some part. But, this also included the time needed to save the results. As the deformation of both meshes needs to be saved, this can take up a significant amount of time—especially due to the higher resolution electrophysiological mesh.

#### 2.3.4. Initialization

Our 3D model of the heart was based on MRI data obtained in diastatic state, meaning in the presence of respective stresses. For initialization, the geometry of an unloaded state had to be determined. Thus, the goal was to define a mesh state which, when end-diastatic pressure is applied, converges toward the initially obtained mesh.

Different from Gerach et al. (2021), we used an approach aiming to find said unloaded state by applying a force to the first and fifth element of the second Piola–Kirchhoff stress tensor ***S***. Looking at the previously defined myocyte coordinate system this meant adding a force in main (f) and in sheet (s) myocyte direction, thus inverting the stretching effect of the diastatic pressure:


(2) $$\begin{array}{r} S=S+\left(\begin{array}{ccc} T_{\text{init}} & 0 & 0\\ 0 & T_{\text{init}} & 0\\ 0 & 0 & 0 \end{array}\right) \end{array}$$

Of course this requires that transmural and the sheet-normal myocyte direction align—if they do not, a rotation matrix would need to be defined. The applied force *T*_init_ was then iteratively optimized, so that the residual left ventricular reference volume (*V*_0_) was compliant with the data from Klotz et al. (2006):


(3) $$\begin{array}{r} V_{0}\approx V_{\text{diastatic}}\cdot \left(0.6-0.006\cdot P_{\text{diastatic}}\right), \end{array}$$

with *V*_diastatic_ being the volume of the segmented diastatic geometry, and *P*_diastatic_ the diastatic pressure). After the residual volume was reached, end-diastatic pressure was applied and the factor ε of interaction between the heart and its surrounding tissue was linearly increased within a time span of 50 ms from 0 kPa to 20 kPa, see Supplementary Figure S1. The ventricular volumes were then given another 250 ms to converge toward their end-diastatic volume.

## 3. Results

### 3.1. Validation of Electrophysiology

**Figure 4:**
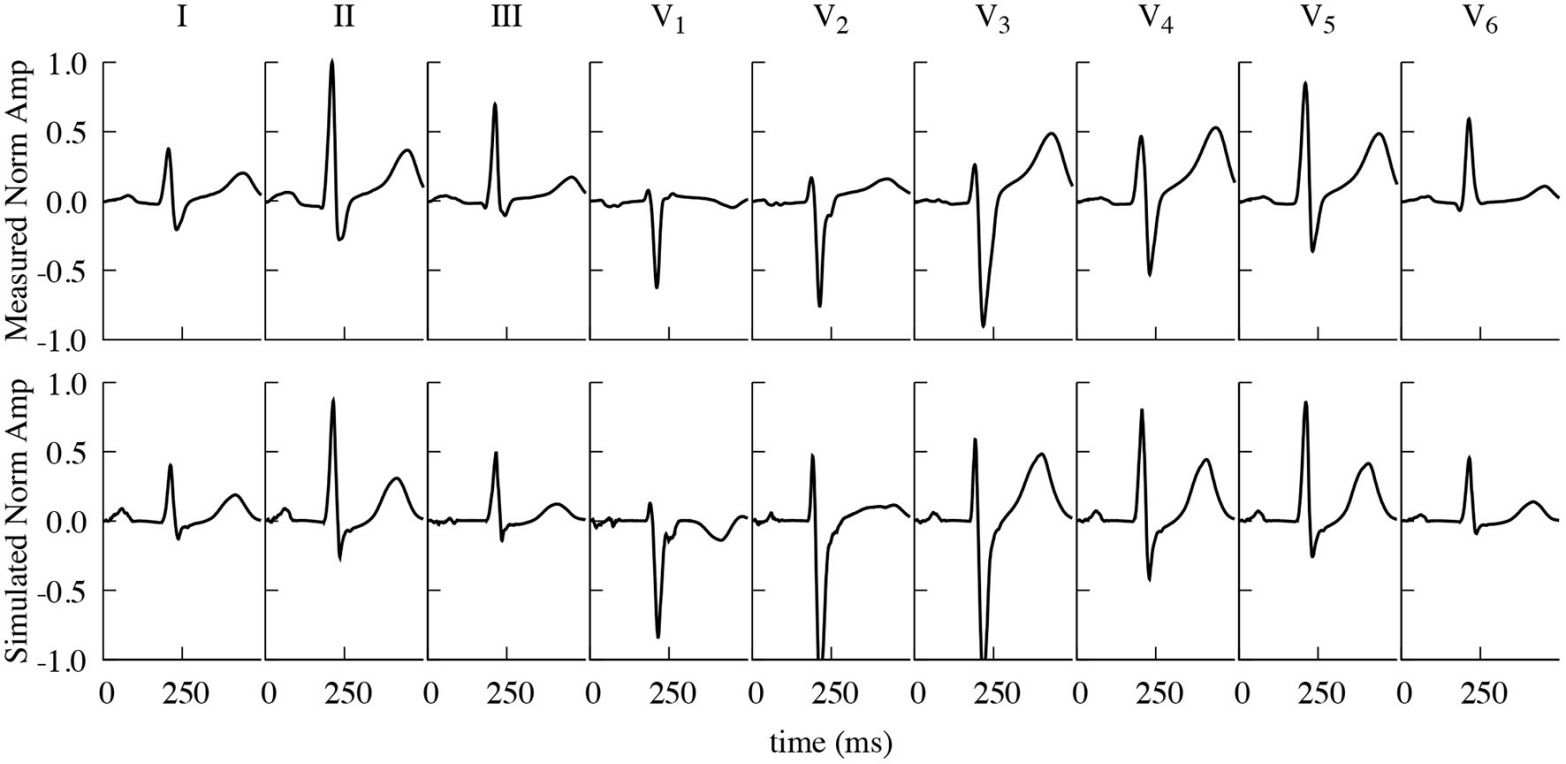
Comparison of measured and simulated ECG traces. Measured (top) and simulated (including contraction) (bottom) Einthoven and Wilson traces, normalized to the R-Peak of Einthoven II. Measured and simulated ECG traces show a strong correlation (Pearson's *r*), averaging 0.872 over all here displayed individual leads.

Figure 4 shows the resulting simulated ECG traces from the dynamic configuration, i.e., the fully coupled electro-mechanic simulation, in comparison to the measured ones, obtained from the same volunteer the geometry was based on, both being normalized to the R-peak of the Einthoven II lead. All simulated leads show very similar morphologies to the measured ones. The average cross correlation factor (Pearson's *r*) between the respective normalized measured and simulated traces that are shown in Figure 4 is 0.872, with the average correlation factor for the three Einthoven leads being 0.882 and 0.867 for the six Wilson leads. The largest difference can be observed in the QT-Time (QT-Time calculated as the time between first ventricular stimulation and the intersection of the tangent in the point of steepest descent with the time axis), with the QT-Time of the simulated traces being on average 32 ms shorter than the measured ones. (I: −28 ms, II: −33 ms, III: −40 ms, V_1_: −20 ms, V_2_: 8 ms, V_3_: −45 ms, V_4_: −50 ms, V_5_: −50 ms, V_6_: −27 ms).

### 3.2. Validation of Mechanics

#### 3.2.1. Initialization

By applying an initial unloading stress of 30 kPa according to Equation (2), the ventricular volume decreased from 142 to 146 mL (as segmented in the end-diastatic state) to 84 mL/89 mL (left/right), which is in concordance with Equation (3) (approximate target residual volume for the left ventricle: 78 mL; right ventricle: 81 mL). Succeeding the unloading stress, diastatic ventricular pressures (determined from previous simulations as convergence values) of 7.3 mmHg for the left side and 6.5 mmHg for the right side of the heart were applied. This resulted in initialized diastatic volumes of ≈152 mL (left ventricle) and ≈156 mL (right ventricle). No further adaptation of stiffness parameters was deemed necessary as these volumes only slightly exceeded the expected (segmented) values.

It should be noted that without interaction with the pericardium, the left ventricular volume showed similar characteristics as with interaction. Yet, the atrial and right ventricular volumes surpassed their segmented diastatic volume (exceeding a volume of 180 mL in case of the right ventricle) illustrating the importance of modeling the pericardial interaction. Additionally, it should be pointed out that the initialized end-diastatic volumes further changed during the course of multiple heart beats, converging toward a left ventricular end-diastatic volume of 136 and 168 mL after three heart beats.

#### 3.2.2. Deformation and Circulation

**Figure 5:**
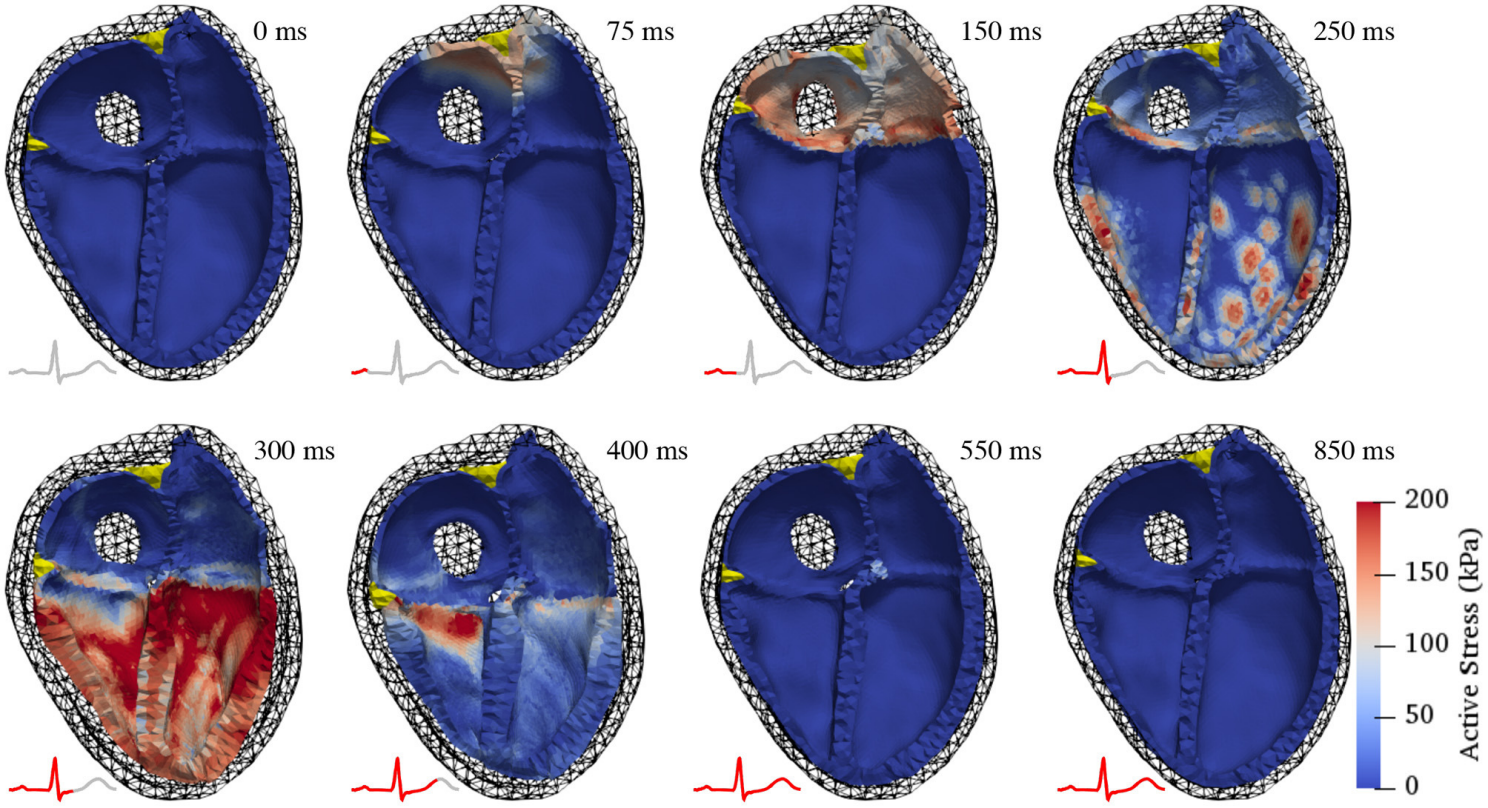
Deformation during a complete cardiac cycle. At first, the atria contract, pulling the atrio-ventricular valve plane upwards and increasing the volumes in the ventricles (150 ms). Subsequently, the ventricles contract isovolumetrically causing a rapid pressure increase (250 ms). Once the ventricular pressure surpasses the respective arterial pressure in the outflow vessels, the ejection phase begins (300 ms), characterized by noticeable thickening of the ventricular walls. Ventricular pressure then drops as the ventricles start relaxing, initially without changes in volume (400 ms). After ventricular pressure has decreased below atrial pressure, the ventricles relax back to their initial state and passively re-fill to 85% of end-diastolic volume during early diastasis (550 and 850 ms). Color coding shows active stress (the force generated by contracting cardiac myocytes). Juxtaposed ECG traces show the temporal relationship of the displayed contraction states with electrical activity.

**Figure 6:**
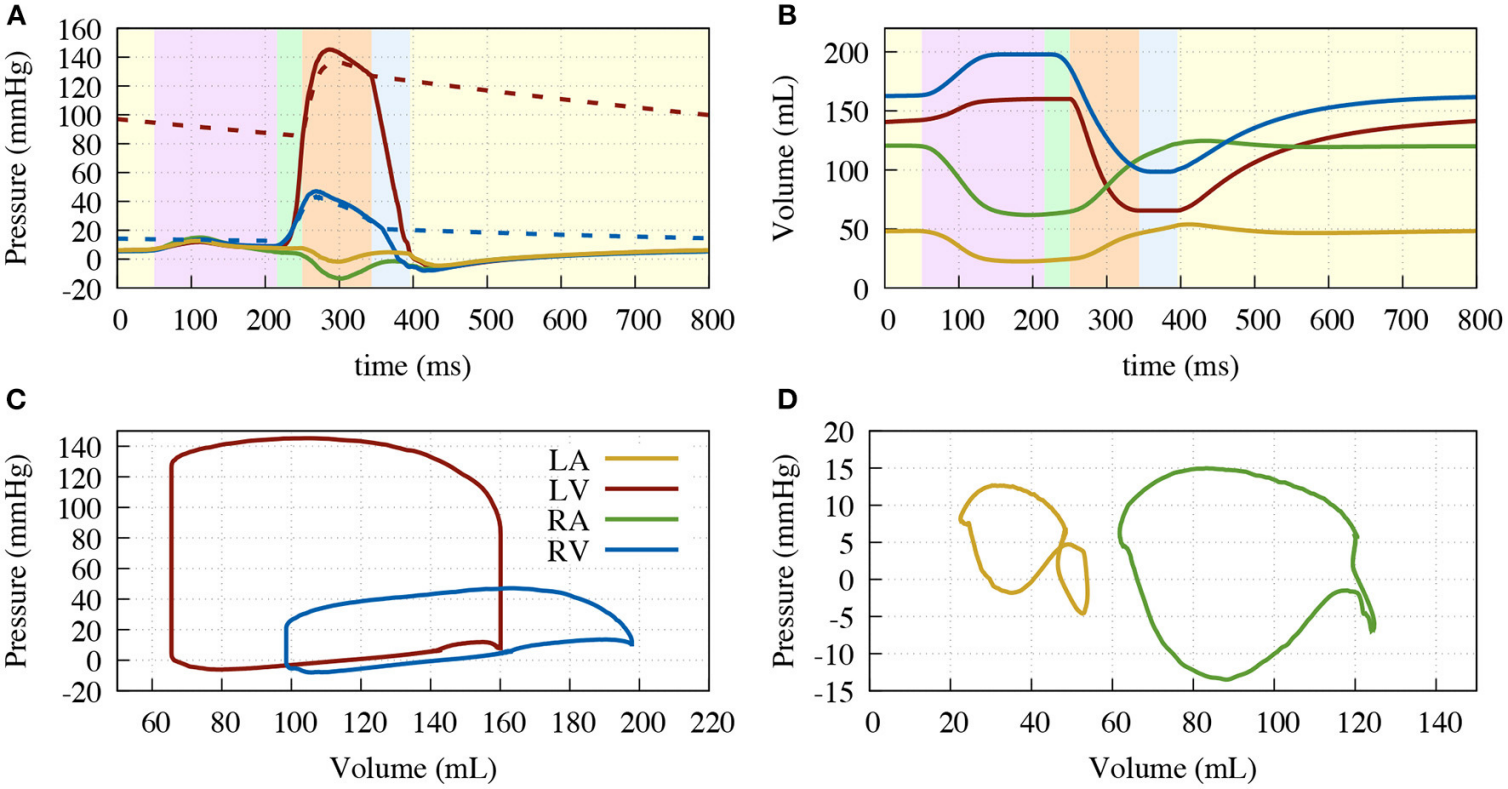
Resulting traces of the circulatory system. **(A)** Pressure over time; **(B)** Volume over time; **(C)** Pressure volume diagram of the ventricles; **(D)** Pressure volume diagram of the atria. Left ventricle (solid red), aorta (dashed red), right ventricle (solid blue), pulmonary artery (dashed blue), right atrium (green) and left atrium (yellow). Background color in **(A,B)** depicting the different phases of the cardiac cycle: atrial contraction (light-purple), isovolumetric contraction (light-green), ejection (light-orange), isovolumetric relaxation (light-blue), relaxation (light-yellow).

Figure 5 shows the resulting deformation and Figure 6 the corresponding pressure and volume traces. Additionally, in more detail, a complete cardiac cycle can be seen in Supplementary Video 1. The simulated heart beat shows all major characteristics expected from a physiological circulatory point of view. First, during the atrial contraction phase (0–150 ms), the atria contract, leading to an increase in ventricular volume by ≈11/21% (left/right) (Figure 6). This is followed by the isovolumetric contraction phase of the ventricles, marking the beginning of systole (225–250 ms). Here, the pressure increases rapidly until it surpasses the “diastolic” aortic or pulmonary artery pressure (85/13 mmHg respectively). Subsequently, during the ejection phase (250–350 ms), the ventricles contract, exhibiting noticeable wall thickening, and eject 58/49% of their end-diastolic content. Once the individual ventricular pressure drops below the “systolic” arterial pressure (125/20 mmHg) of the respective outflow vessel, but is still above the atrial pressure, isovolumetric relaxation takes place (350–400 ms), indicating the end of the systole and the beginning of the diastole. After ventricular pressure drops below the atrial pressure, the ventricles relax back to their initial end-diastatic state (400–800 ms).

#### 3.2.3. Rotation and Valve Plane Movement

**Figure 7:**
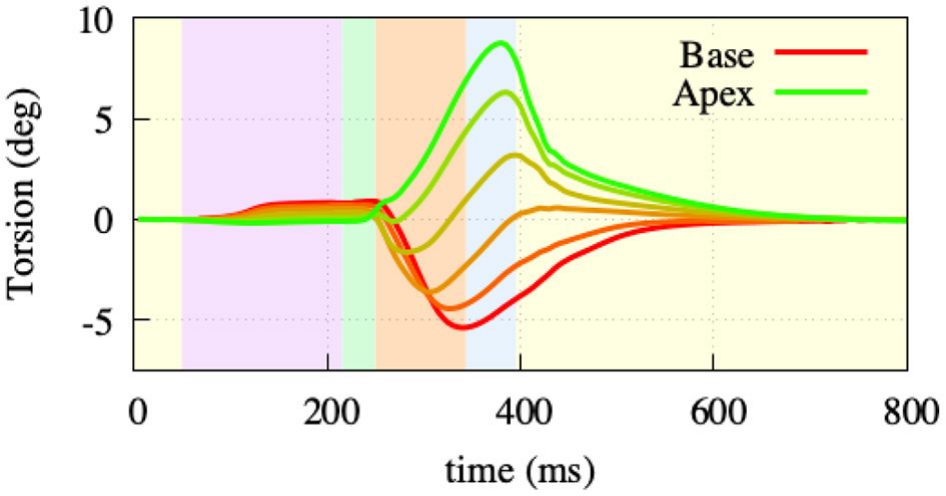
Twisting motion measured at 5 points of the left ventriclular free wall. During atrial contraction (light-purple) and isovolumentric contraction (light-green), the apex shows a clockwise and the base a counterclockwise rotation. Throughout the ejection (light-orange) phase, the rotational direction switches to a counterclockwise rotation in the apex and clockwise rotation in the base. Afterwards, both base and apex go back to their initial state as part of the relaxation.

To assess the occurring displacement during the heart cycle, both the rotational movement and the atrioventricular plane displacement (AVPD) were further assessed. Rotation was measured at 5 points of the endocardial side of the left ventricular free wall (Figure 7). The overall twisting motion is hereby in concordance with published data from Sengupta et al. (2008) in terms of characteristic and amplitude during the different contractile phases. During atrial contraction, there is a small clockwise (viewing from base toward apex) rotation in the base and a minor counterclockwise rotation in the apex. During isovolumetric contraction, this rotation then starts to flip to a rapid counterclockwise rotation in the base and a clockwise rotation in the apex. The peak twisting angle of −5° in the base and 9° in the apex is then reached amid the ejection phase (−5° and 10° in the measured data). Subsequently, during relaxation, the ventricle rotates back in two phases (fast/slow), a characteristic which can also be observed in the data measured by Sengupta et al. (2008).

**Figure 8:**
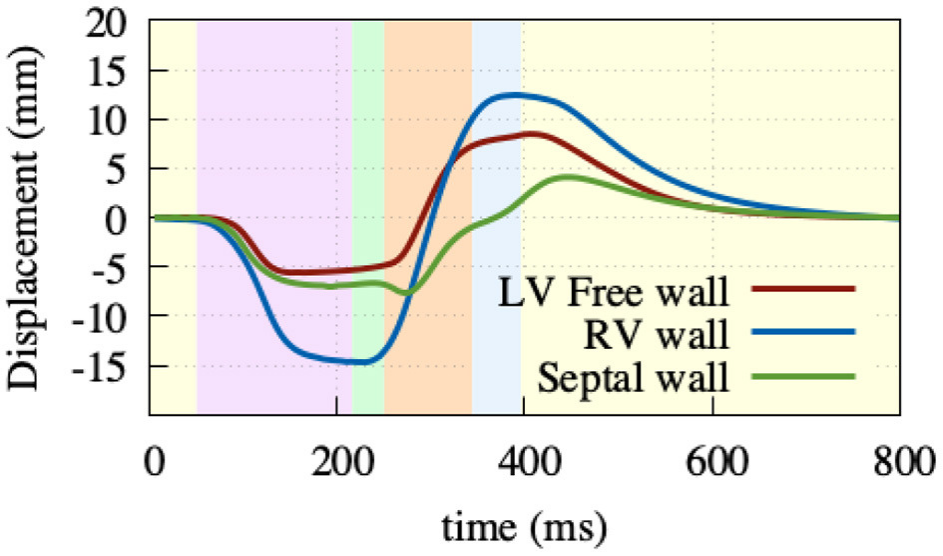
Atrioventricular plane displacement. Extracted at the base of the right ventricular free wall (blue), base of the left ventricular free wall (green), and base of the septal wall (red), with positive displacement indicating a shift toward the apex. At the beginning of the heart cycle during atrial contraction (light-purple), the valve plane is pulled upward to then rapidly move downwards during ventricular contraction (light-green, light-orange), and then back to its initial state during the relaxation phase (light-yellow).

AVPD was extracted at three points: at the base of the left ventricular free wall, at the base of the right ventricular free wall, and at the base of the septal wall, see Figure 8. Positive displacement represents a movement of the valve toward the apex and negative toward the top of the atria. Due to the atrial contraction, the valve plane first moves upwards by –6/–7/–15 mm (LV/septum/RV) to then move downwards during ventricular ejection by 14/11/27 mm. The resulting traces are comparable to reported data from Maffessanti et al. (2013), Fritz et al. (2014), and Pfaller et al. (2019). Unfortunately, as the original Cine-MRI data-set from Keller et al. (2011) was recorded solely in short-axis view, we could not validate against the volunteer's AVPD.

### 3.3. Impact of Deformation on the ECG and BSPM

To exclude possible effects on the action potential that *strong coupling* has at the single cell level, we used the same approach as in Smith et al. (2003). Thus, we mapped the resulting membrane voltage transients of the dynamic configuration to one single static geometry at a specific time point during the cardiac cycle. The time points for the three presented static cases are the end-diastatic (Figure 5, 0 ms), end-diastolic (150 ms), and end-systolic (400 ms) states of the heart.

**Figure 9:**
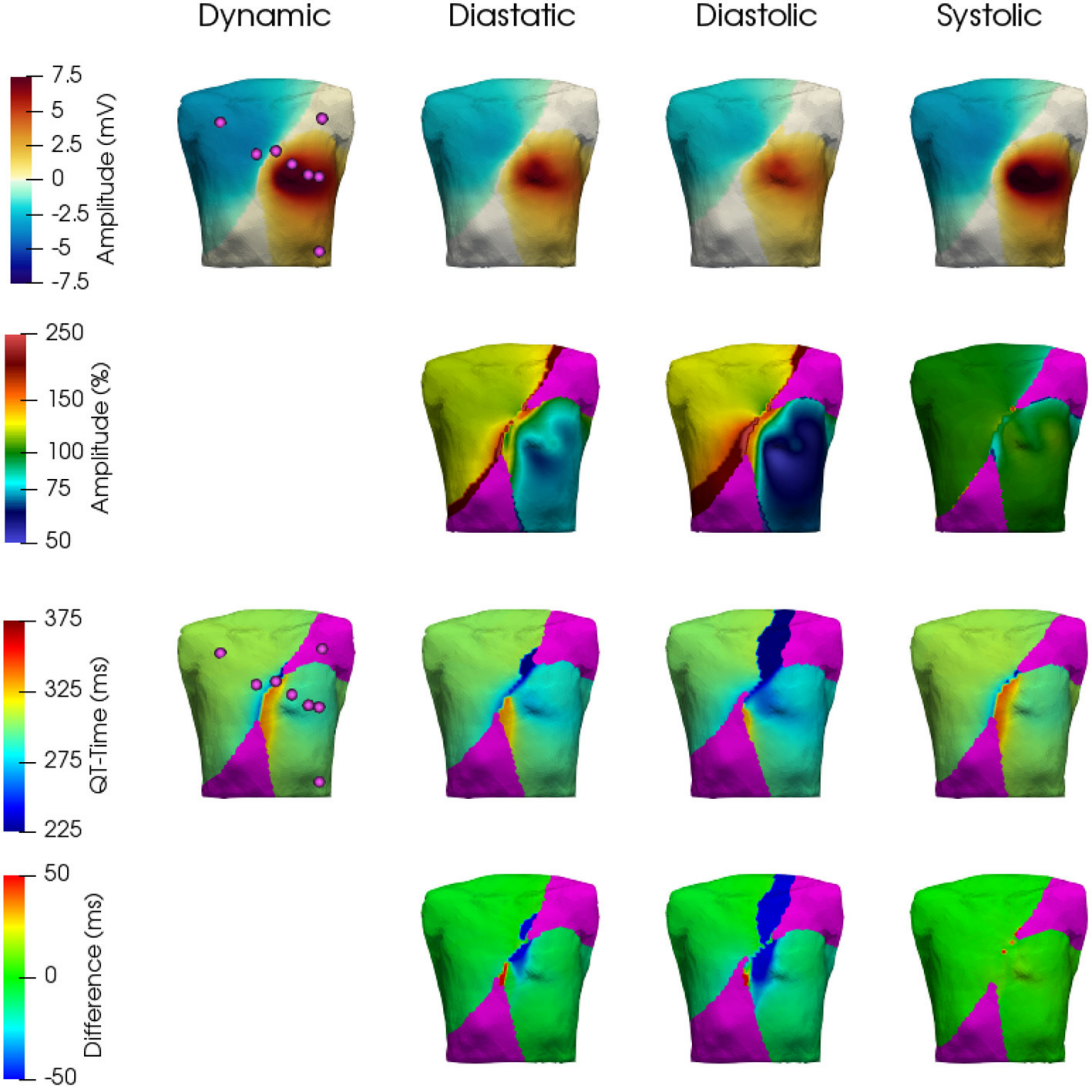
Comparison of resulting T-Wave amplitudes and durations. Top row: T-Wave amplitudes extracted from the BSPM for the four cases—dynamic, diastatic, diastolic, systolic. 2nd row: T-Wave amplitudes in % with respect to the dynamic case. 3rd row: T-Wave extracted from the BSPM. 4th row: Difference in ms of the three static cases with respect to the dynamic one. The area shown in Magenta was ignored, as the absolute T-Wave amplitude here was below 0.5 mV.

In general, and as expected, it can be observed that the three static cases show the highest similarity to the dynamic configuration, when aligning with their respective contractile state. Thus, during atrial and the beginning of ventricular depolarization, the end-diastatic and end-diastolic cases show the highest similarity to the dynamic model. As the heart cycle progresses, differences between the early static cases and the dynamic configuration emerge and the end-systolic case displays the highest similarity. This holds also true for the biggest relative differences on the BSPM, which can be observed during the later stages of the cardiac cycle, especially comparing early static cases with the dynamic configuration. Supplementary Video 2 shows the different resulting BSPMs dynamics, as well as the respective differences between individual cases. Figure 9 shows the resulting T-Wave amplitudes and T-Wave duration differences in more detail. The most prominent difference is the increased amplitude near the apex of the ventricles in the dynamic and systolic cases, most likely due to the rotational movement.

**Figure 10:**
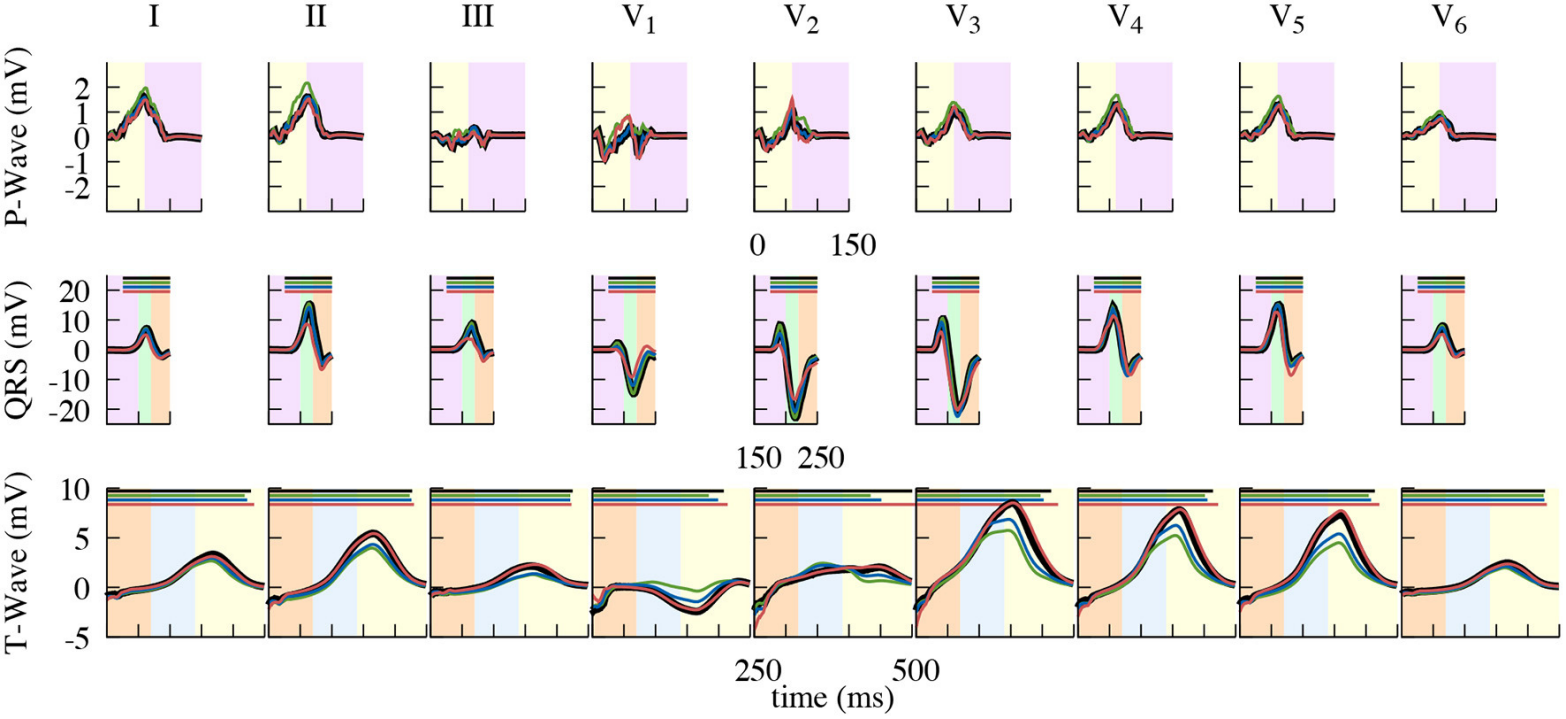
Close up of the resulting standard lead ECG traces. Comparison of using the dynamic (black), end-diastatic (green), end-diastolic (blue), end-systolic (red) configuration to calculate different clinical ECG leads. Shown is the impact on the P-Wave (top), the QRS-Complex (middle), and the T-Wave (bottom). Background color depicting the different phases of the cardiac cycle: atrial contraction (light-purple), isovolumetric contraction (light-green), ejection (light-orange), isovolumetric relaxation (light-blue), relaxation (light-yellow). QT-Time, also shown in Table 2, is depicted as a bar on top in the respective color.

These differences also reflect in the clinically used leads Einthoven I–III and Wilson V_1_–V_6_, which can be seen in Figure 10 (electrode positions shown in Figure 9). The largest influence of contraction can be observed in the leads derived closest to the ventricles, i.e., Wilson V_3_, V_4_, and V_5_ during the ejection, isovolumetric relaxation, and early relaxation phases. In terms of the P-Wave, there is only a small difference in lead V_2_ between the end-diastatic, end-diastolic, end-systolic, and deforming configurations (top row Figure 10). The QRS-Complexes of the static cases show some differences (middle row of Figure 10). Both end-diastatic and end-diastolic cases show close to no difference in R and S-peak, but the end-systolic case displays differences in all standard leads, with an overall decrease in R-Peak amplitude.

Regarding the T-Wave, the end-systolic configuration shows almost no differences, yet nearly all leads of the end-diastatic and end-diastolic cases differ in amplitude (Table 1 and bottom row of Figure 10). With respect to the dynamic configuration, both of these early configurations show distinct differences in maximal T-Wave amplitude, with reductions by up to 40%. Out of the three Einthoven leads, lead (II) shows the biggest difference in amplitude. The Wilson leads closest to the heart (V_3_–V_5_) show the largest differences in absolute amplitude between the dynamic and the early static cases, while V_2_ shows the biggest relative difference in amplitude. This also applies when comparing the two early static cases to one another, with the biggest absolute amplitude difference in V_3_–V_5_ and the biggest relative difference in V_1_. These differences also manifest themselves in the QT-Time (Table 2). Concordantly, leads that show a high difference in amplitude also exhibit differences at the end of T-Wave. The strongest impact can be observed on V_2_, where a difference in morphology leads to the biggest shift in the QT-Time.

**Table 1:** T-Wave amplitude of the dynamic configuration in the standard leads and the respective differences of the three static cases in respect to the dynamic one.

**Lead**	**Dynamic (mV)**	**End-diastatic (%)**	**End-diastolic (%)**	**End-sytsolic (%)**
I	3.30	80	88	93
II	5.30	72	79	99
III	2.15	60	64	107
V_1_	2.43	73	59	93
V_2_	2.00	119	100	102
V_3_	8.60	67	80	99
V_4_	7.80	67	80	100
V_5_	7.30	61	73	105
V_6_	2.40	81	87	96

*Dynamic T-Wave amplitude/respective end-diastatic, end-diastolic, and end-systolic amplitude*.

**Table 2:** End of T-Wave (measured from earliest ventricular activation) in the dynamic configuration in the standard leads and differences of the three static cases in respect to the dynamic one.

**Lead**	**Dynamic (ms)**	**End-diastatic (ms)**	**End-diastolic (ms)**	**End-sytsolic (ms)**
I	303	–10	–6	5
II	302	–4	–1	3
III	300	–4	–4	–2
V_1_	283	–24	–9	6
V_2_	334	–75	–58	7
V_3_	289	–17	–12	11
V_4_	289	–13	–9	8
V_5_	289	–10	–6	7
V_6_	302	–2	–1	3

*End of dynamic T-Wave, difference of end-diastatic, end-diastolic, and end-systolic end of T-Wave*.

## 4. Discussion

In this study, we present a fully coupled whole-heart model, including volume exchange with a circulatory model and interaction with the pericardium. We incorporated our model into the torso to investigate effects on ECG parameters that arise due to cardiac contraction—effects that are neglected in static models of cardiac electrophysiology.

Our simulated “dynamic heart” ECG traces show a strong correlation with measured data, except for prominent differences related to the time of repolarization in the ventricles and thus the QT-Interval. The cause for this can be traced back to the decision to initialize the used OVVR cell model with a heart rate of 70 bpm, which—on a single cell level—results in an APD of > 270 ms.

On the mechanical side, we focused on our model performing physiologically with respect to circulation, initialization, AVPD, and ventricular torsion. For the circulation, our fully coupled closed-loop lumped model of the complete circulatory system reproduced all major features of pressure and volume dynamics (cf. Figure 6. In terms of AVPD and torsion (cf. Figures 7, 8), the model recreated published behavior (Sengupta et al., 2008; Maffessanti et al., 2013; Fritz et al., 2014; Pfaller et al., 2019).

Regarding the impact of cardiac motion on the ECG, the biggest relative difference in the respective clinically prevalent leads can be observed at maximal contraction, i.e., during the ejection phase. Here, the T-Wave as a clinically used ECG feature coincides with significant deformation (i.e., wall thickening and rotation, cf. Figures 5–7, 10 at 300–400 ms). One could potentially even argue that the Wilson leads are placed one intercostal space too high in our model and thus the effects on these respective leads would be even higher (see 1st row of Figure 9). A shift of the Wilson leads further down on the torso would increase the amplitude in all leads, except for V_1_ – where one could potentially see a flip in T-Wave polarity. Additionally, the impact of cardiac contraction on V_3_–V_5_ would increase (see 2nd row of Figure 9). From the pattern of difference in ECG signal amplitude, we hypothesize that overall the Einthoven leads are predominantly influenced by AVPD, while the Wilson leads, being located closer to heart, are more strongly affected by the rotational movement of the ventricles.

Interestingly, in comparison to work by Keller et al. (2011), which was based on the same volunteer, our results show a different impact. Their main finding was a decrease in Einthoven II T-Wave amplitude of up to 40% when using a dynamic configuration, and a further not specified higher impact in the area of the heart. Their findings of a decrease in Einthoven II T-Wave amplitude, contradict the findings of our work as we observed an increase of 12% in the respective lead. In comparison to our model, the deforming geometry used by Keller et al. (2011) (based on the segmentation of a Cine-MRI data-set) does not exhibit rotational movement, minimal APVD, and a LV ejection fraction of only 25%, see Supplementary Video 3. Unfortunately, their simulated membrane voltages were not saved with the geometry and could not be consulted for comparison. In an attempt to reproduce their findings, we used the consistent biventricular coordinates system *Cobiveco* as published by Schuler et al. (2021) to map our calculated membrane voltages to their deforming geometry. We then used what we presume was the pipeline used by Keller et al. (2011) to do the forward calculation. The resulting ECG did not show any of the characteristics reported by Keller et al. (2011), but rather displayed similar, yet less pronounced, differences as we have shown with our model.

Rather small impact on the Einthoven leads were also reported in Wei et al. (2006), who also used the approach of creating a deforming geometry based on a MRI data-set. But also here, our model contradicts their findings of a decreased amplitude in the closely located Wilson leads. This could again be explained by a lack of rotational movement, as Wei et al. (2006) did not investigate whether the rotational movement of their segmented geometry was in a physiological range. As their technique used to create the dynamic mesh is similar as to that employed by Keller et al. (2011), it can be argued that it suffers from similar shortcomings. The impact as described on V_2_ in the work by Smith et al. (2003) could be seen similar to the one in this work, if one would move the V_2_ electrode slightly on the transverse plane.

When looking at the three non-deforming cases in relation to the dynamic configuration, they are each able to represent their respective contractile state, but differences appear when compared to other parts of the cardiac cycle. The end-diastatic and end-diastolic case show minimal differences to one-another, and to the dynamic case during atrial and ventricular depolarization. Maximal differences are seen when compared to the end-systolic case and to the dynamic model during ventricular repolarization. Accordingly, the end-systolic case faithfully represents later stages of the cardiac cycle, and shows the greatest differences during the earlier stages.

Based on the presented observations from our model we conclude that the impact of cardiac contraction on the ECG should not be overlooked—especially, when measured BSPM are used to parameterize action potential repolarization characteristics. This does not mean that any ECG calculated on a static end-diastolic mesh, as it is normally the case, should be discarded, but one should be aware of potential errors in T-Wave amplitude and QT-Time. This is especially true when looking at pathologies which alter APD (e.g., Long or Short QT-Syndromes), affect the contractile function (i.e., fibrosis), affect the temporal relation between repolarization and contraction (e.g., 2:1 blocks, bundle branch blocks, and other arrhythmias), or when attempting to solve the inverse problem of ECG (ECG imaging) with a focus on repolarization. Our results also show that, in theory, one could avoid the associated computational cost of a dynamic electro-mechanical set-up by using an end-diastatic geometry to investigate the P-Wave, an end-diastolic to investigate the QRS-Complex, and an end-systolic cardiac deformation state to investigate the T-wave.

### 4.1. Points of Improvement and Future Work

The specific impact shown within the scope of this work is dependent on the used geometry. Even though we are able to show that cardiac contraction can have a significant impact on parts of the ECG, in order to obtain a more generalized description, a broader study would be needed. One way could be to use the cohort of four-chamber meshes as published by Strocchi et al. (2020a). Yet, as these meshes are solely depicting the heart itself, a solution for the missing torso mesh would need to be found. The work by Nguyên et al. (2015) and Mincholé et al. (2019) showed that heart position and size can have an impact on the duration and amplitude of the QRS Complex and T-Wave. But these alterations result in a constant change in the ECG and not a temporal one, as it is the case with the impact of contraction. This only increases the importance of the aforementioned use of at least a static systolic representation of the heart when looking at electrophysiological repolarization properties.

In terms of electrophysiology and ECG, the resulting traces from the simulations could be further improved. For atrial activation, optimizing the electrical conductivity could lead to a more physiological excitation pattern and thus P-Wave. Yet, as the difference between the diastatic and diastolic configuration were marginal, this would not alter the results shown in regard to the impact of cardiac contraction on the ECG. Further, extracellular conductivities could be scaled as to obtain comparable amplitudes and eliminate the need of normalization to compare measured and simulated traces. But, to obtain comparable data to Keller et al. (2011), this was not done within the scope of this work. Additionally, as stated, the difference in QT-Interval in the leads could be reduced by either pacing the OVVR cell model at a different frequency, or by modifying selective parameters to obtain better matching APD.

For the mechanical part, some of its limitations also arise from the electrophysiology. The AVPD could probably be improved, if the active stress component in the atria would be zero during the ventricular ejection phase, consequently not counteracting the initial downward pull of the ventricles. Additionally, the initialization showed that using one set of parameters describing the passive properties of all cardiac cells might not be valid. Adapting those parameters for each cavity of the heart would be attractive, but data is currently sparse. Further, our validation was based on literature values. A better, subject-specific validation would be conceivable using high-resolution CINE MR imaging with displacement encoding with stimulated echoes (DENSE) to track ventricular wall movements (such as AVPD and rotation) (Aletras et al., 1999; Carruth et al., 2021).

Regarding future work, it would be interesting to investigate the impact of cardiac contraction on the ECG even further. The implementation of pathophysiologies such as Short or Long QT-Syndrome, which alter the temporal relationship of repolarization and the contraction-cycle, could create new insights into links between mechanical contraction and T-Wave morphology. Especially, the latter could yield interesting results, as the delayed repolarization would move to a later stage in the contractile cycle, thus reducing the impact of cardiac contraction.

Additionally, the potential impact of tissue deformation on the electrical conductivity tensor **σ** (Sachse et al., 2000; McNary et al., 2008) was ignored within the scope of this work. In the healthy case, this would probably affect ventricular repolarization and therefore the T-Wave, as deformation at this point is largest. But especially in pathological cases, it would be important to use a mechanistic model including complex electro-mechanical interactions, as all components are interdependent. When looking at ventricular arrhythhmias, an altered electrophysiological behavior could have knock-on effects on the mechanics, which in turn could alter the underlying electrophysiological properties, potentially resulting in a feedback loop. Similarly, changed mechanical properties (e.g., due to myocardial scarring from infarction) may have an influence on electrophysiology. Such cardiac mechano-electric feedback is well-established based on cellular observations (Kohl et al., 1999; ter Keurs, 2011; Pfeiffer et al., 2014). The suggested effects on electrical arrhythmias, however, have in computational models so far only been reproduced in simplified electro-mechanical models of cardiac tissue (e.g., Colli Franzone et al., 2017; Sahli Costabal et al., 2017). Our model of the complex electro-mechanical interactions may therefore be useful to relate or extrapolate experimental *in vitro* results of mechano-electric feedback to the whole heart or ECG.

## Data Availability Statement

The original contributions presented in the study are included in the article/Supplementary Materials, further inquiries can be directed to the corresponding author.

## Author Contributions

RM: investigation and writing and visualization of original draft. RM, EW, and SS: methodology. GS and AL: project administration. GS: resources and funding acquisition. RM and GS: conceptualization. RM, EW, SS, AL, and GS: review and editing. All authors contributed to the article and approved the submitted version.

## Funding

RM and GS gratefully acknowledge financial support by the Deutsche Forschungsgemeinschaft (DFG, #394630089). EW and GS gratefully acknowledge financial support by the DFG (#183027722). RM, EW, and GS are members of the Collaborative Research Centre SFB 1425 of the DFG (#422681845). AL gratefully acknowledges financial support by the European Metrology Programme for Innovation and Research [18HLT07 MedalCare]. AL is member of the Collaborative Research Centre SFB 1173 of the DFG (#258734477). The article processing charge was funded by the Baden-Wuerttemberg Ministry of Science, Research and Art and the University of Freiburg in the funding programme Open Access Publishing.

## Conflict of Interest

The authors declare that the research was conducted in the absence of any commercial or financial relationships that could be construed as a potential conflict of interest.

## Publisher's Note

All claims expressed in this article are solely those of the authors and do not necessarily represent those of their affiliated organizations, or those of the publisher, the editors and the reviewers. Any product that may be evaluated in this article, or claim that may be made by its manufacturer, is not guaranteed or endorsed by the publisher.
