# Supplementary material for: A Fully-Coupled Electro-Mechanical Whole-Heart Computational Model: Influence of Cardiac Contraction on the ECG
Source: Front Physiol. 2021 Dec 16;12:778872. doi: 10.3389/fphys.2021.778872 (PMC8716847; doi:10.3389/fphys.2021.778872)
Supplement: Supplementary file 4 [file Data_Sheet_1.pdf]

# Supplementary Material

## 1 SUPPLEMENTARY METHODS

### 1.1 Electrophysiology

#### 1.1.1 Numerical Methods for the Monodomain Equation

Simulation of electrical excitation and wave propagation across the tissue were calculated as part of our electro-mechanical simulation framework (Gerach et al., 2021). The electrophysiological part is hereby based on the verified framework *acCELLerate* (Seemann et al., 2010; Niederer et al., 2011). Thus, as published, the transmembrane voltage $V_\mathrm{m}$ across the tissue was governed by the monodomain model, a reaction–diffusion equation:

$$\nabla \cdot (\boldsymbol{\sigma} \nabla V_\mathrm{m}) = \beta \left( C_\mathrm{m} \frac{\partial V_\mathrm{m}}{\partial t} + I_\mathrm{ion} \right) \tag{S1}$$

with $\boldsymbol{\sigma}$ the conductivity tensor, $\beta = 140,000\,\mathrm{m}^{-1}$ the myocyte surface-to-volume ratio, $C_\mathrm{m} = 0.01\,\mathrm{F/m^2}$ the cell membrane capacitance, and $I_\mathrm{ion}$ the accumulation of ion currents across the membrane (i.e., currents between intra- and extracellular space), calculated using different myocyte electrophysiology models.

The conductivity tensor $\boldsymbol{\sigma}$ comprises a longitudinal ($\sigma_\ell$, conductivity along myocyte direction) and transverse ($\sigma_\mathrm{t}$) component. These components were chosen such that physiological conduction velocities (CV) of $800$ mm/s along myocyte orientation and $550$ mm/s in the transverse directions were achieved (as in Keller et al. (2011)). Optimizing $\boldsymbol{\sigma}$ accordingly in a simple 'stick' geometry, composed of tetrahedra of the same average edge length as the heart mesh, resulted in $\sigma_\ell$ = 0.215 S/m and $\sigma_\mathrm{t}$ = 0.095 S/m (anisotropy factor between longitudinal and transverse direction of 2.65). The same conductivity values were used for atrial and ventricular tissue.

The finite element method was used to spatially discretize the monodomain equation on the tetrahedral mesh of the heart. The Crank-Nicolson method was used for temporal discretization and the problem was solved in a time-stepping manner (with $\Delta t = 50\,\mu\mathrm{s}$). The mathematics behind our discretization methods are fully documented in Gerach et al. (2021). Initial values for $V_\mathrm{m}$ were locally assigned from the respective myocyte models which are detailed below.

The open-source linear algebra toolkit *PETSc* was used to solve the discretized equation for every time step, using the Gauss-Seidel method for preconditioning and the minimum-residual method (*MINRES*) as Krylov-subspace solver (Paige and Saunders, 1975; Balay et al., 2021). A time step was considered converged if the residual error of the iteration was below $10^{-8}$ or if the relative change in residual error was below $10^{-12}$.

Our scheme made use of the full mass matrix and evaluated ion currents at tetrahedral nodes, which has been shown to produce low errors in CV even at 'moderate' spatial resolutions such as ours (Pezzuto et al., 2016, cases 'LHS full, RHS interpolated, full' and '$\theta_\mathrm{lhs} = \theta_\mathrm{rhs} = 0$').

#### 1.1.2 Forward Problem

For each time step, the forward problem posed by

$$\nabla \cdot \left( \left( \boldsymbol{\sigma}_\mathrm{i} + \boldsymbol{\sigma}_\mathrm{e} \right) \nabla \Phi_\mathrm{e} \right) = -\nabla \cdot \left( \boldsymbol{\sigma}_\mathrm{i} \nabla V_\mathrm{m} \right) \tag{S2}$$

was solved for $\Phi_\mathrm{e}$, the extracellular potentials at each mesh node. Intra- and extracellular conductivity tensors ($\boldsymbol{\sigma}_\mathrm{i}$ and $\boldsymbol{\sigma}_\mathrm{e}$, respectively) were assigned organ-specific values (supplementary table S1). Our model includes conductivities for all organs (except for skeletal muscle which could not be segmented from MRI) that were previously identified as having large influences on the ECG (Keller et al., 2010).

The discretized equation was solved using *PETSc* with symmetric Gauss-Seidel preconditioning and *MINRES* as Krylov-subspace solver. Convergence criteria were an absolute residual value below $10^{-50}$ or a relative residual improvement of less than $10^{-10}$.

**Table S1.** Tissue conductivities used for body surface potential map (BSPM) and ECG calculations. Where applicable, directions *longitudinal* and *transverse* refer to the local myocyte orientation. Other conductivities were treated as isotropic.

| Tissue | Conductivity (S/m) | | Source |
|---|---|---|---|
| Fatty tissue | 0.035 | | Gabriel et al. (1996)[2] |
| Skin | 0.0002 | | |
| Blood[1] | 0.7 | | |
| Lung | 0.03 | | |
| Intestines | 0.01 | | |
| Kidney | 0.05 | | |
| Liver | 0.02 | | |
| Spleen | 0.03 | | |
| Heart | *longitudinal* | *transverse* | |
| *intracellular* | 0.3 | 0.031525 | Colli Franzone et al. (2005) |
| *extracellular* | 0.15 | 0.05 | Gabriel et al. (1996)[2]<br>Keller et al. (2010) (anisotropy) |

[1] Only modelled in the heart cavities.

[2] Extrapolated to 0 Hz using the Cole-Cole equation (Cole and Cole, 1941).

## 1.2 Mechanics

### 1.2.1 Material Law

As described in 2.3.1 the material law by Usyk et al. (2000) is used within this work to describe the passive stress-strain relationship in the ventricles as well as atria. Its energy function is given by:

$$\begin{aligned} &W = c(e^Q - 1) + \frac{\kappa}{2}(\det(\boldsymbol{F}) - 1)^2 \\ &\text{with} \\ &Q = b_\mathrm{ff}E_\mathrm{ff}^2 + b_\mathrm{ss}E_\mathrm{ss}^2 + b_\mathrm{nn}E_\mathrm{nn}^2 + b_\mathrm{fs}(E_\mathrm{fs}^2 + E_\mathrm{sf}^2) + b_\mathrm{fn}(E_\mathrm{fn}^2 + E_\mathrm{nf}^2) + b_\mathrm{sn}(E_\mathrm{sn}^2 + E_\mathrm{ns}^2), \end{aligned} \tag{S3}$$

where $\boldsymbol{F}$ is the deformation tensor and $\boldsymbol{E}$ the Green strain tensor in local myocyte orientation with $\mathrm{f}$, $\mathrm{s}$, and $\mathrm{n}$ referring to the orthonormal main myocyte, sheet, and sheet-normal axes, respectively. The parameters of the material law were set according to Gurev et al. (2015) ($c = 880\,\mathrm{Pa}$, $b_\mathrm{ff} = 6$, $b_\mathrm{ss} = 7$, $b_\mathrm{nn} = 3$, $b_\mathrm{fs} = 12$, $b_\mathrm{fn} = 3$, $b_\mathrm{sn} = 3$, and $\kappa = 10^6\,\mathrm{Pa}$).

For all other types of tissue, the energy function was characterized by using a neo-Hookean material, as proposed by Gerach et al. (2021): Other tissue types were characterized using a neo-Hookean material, as

proposed by by Gerach et al. (2021). Its energy function is given by:

$$W = \mu(\mathrm{tr}(\boldsymbol{C}) - 3) - \mu \ln(\det(\boldsymbol{C})) + \frac{\kappa}{2} \ln^2(\det(\boldsymbol{C})), \tag{S4}$$

with $\boldsymbol{C}$ being the right Cauchy–Green tensor, $\kappa$ being $10^3$ Pa for all tissue types and $\mu$ set to $10^5$ Pa for valves, $3725$ Pa for fatty tissue, $10^4$ Pa for all vessels, and $10^4$ Pa for the surrounding tissue.

### 1.2.2 Surrounding Tissue

As described in 2.3.2, within in this work, we use a friction-less contact problem to emulate the influence of the pericardial sac on the motion of the heart. The used approach is based on the work by by Fritz et al. (2014). For that two surfaces are defined, the first one being the inside of the non-cardial tissue layer surrounding the heart and the other the epicardial surface of the heart. The resulting acting force, in normal direction, on the respective elements of the two surfaces is the regulated by the gap function ($\boldsymbol{g}_\mathrm{N}$). Within the scope of this work the original definition of said gap function, as defined by Fritz et al. (2014) in eq. 24, was replaced with a non-linear continuous function:

$$\boldsymbol{g}_\mathrm{N}(\xi) = e^2\, x(\xi)^2\, e^{-2x(\xi)}\, \boldsymbol{n}^\mathrm{m} \quad \text{with} \quad x(\xi) = \frac{d_{F\max}}{||\boldsymbol{x}^\mathrm{s}(\xi) - \boldsymbol{x}^\mathrm{m}(\xi)||}\,, \tag{S5}$$

where $\boldsymbol{n}^\mathrm{m}$ is the normal of the epicardial surface element, $\boldsymbol{x}^\mathrm{m}$ its projection in normal direction to the closest non-cardiac tissue surface element, $\boldsymbol{x}^\mathrm{s}$ the projection in normal direction of the surrounding tissue surface element to the epicardium, and $d_{F\max} := 1$ cm the distance at which the attraction force is to be maximal (the attraction force will reduce when surpassing this distance as to avoid numerical instability during initialization due to overly large attraction forces). A comparison between the originally proposed gap function by Fritz et al. (2014) and or new one can be seen in fig. 1.2.2.

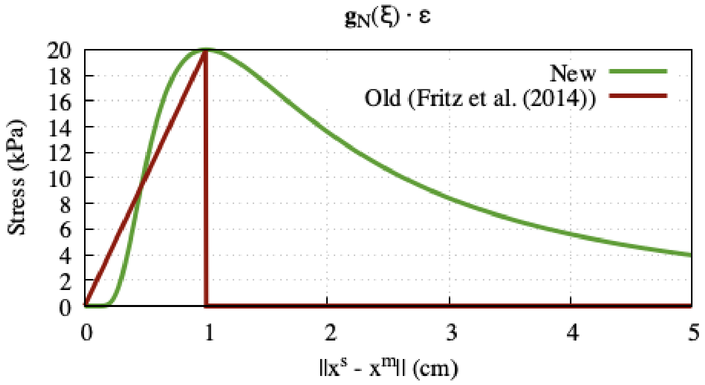


**Figure S1.** Gap function of the frictionless contact problem, multiplied by the maximal attractile force $\varepsilon$, which governs the acting force the epircardial surface of the heart and the inside surface of the surrounding tissue

### 1.2.3 Circulatory system

Within the scope of this work the circulatory system is modeled as a fully coupled closed-loop lumped model, as proposed by Gerach et al. (2021), see 2.3.2 of the main manuscript. Therefore, all distinct parts of the circulatory system are represented by a series of diodes, resistors, and capacitances. see fig. S2.

The finite element mechanics model and the lumped circulation model are then coupled with the goal of iterative adaption of the respective cavity pressures such that the in- and outflows match one another. To avoid accumulation of volume errors over time, the absolute difference in cavity volumes on either side is minimized instead of the difference in incremental volume changes. Therefore, for every time step, the pressure for each cavity is first extrapolated from previous pressures using a fourth order Adams-Bashforth scheme. If the resulting volume differences are not within a certain threshold ($10^{-7}$ mL), the cavities' pressures get perturbed one by one to calculate the compliance matrix of the circulatory system. Using Newton's method, the pressures can then be estimated once more. For all following iterations within one time step, the modified Newton approach from Kerckhoffs et al. (2007) is used, which only perturbs the pressures of the lumped model, so that the compliance matrix of the circulatory system can be updated without having to recompute multiple solutions to the finite element model. The parameters as published by Gerach et al. (2021) were adjusted interactively (see table S2) to fit the different geometry as well as the different heart rate.

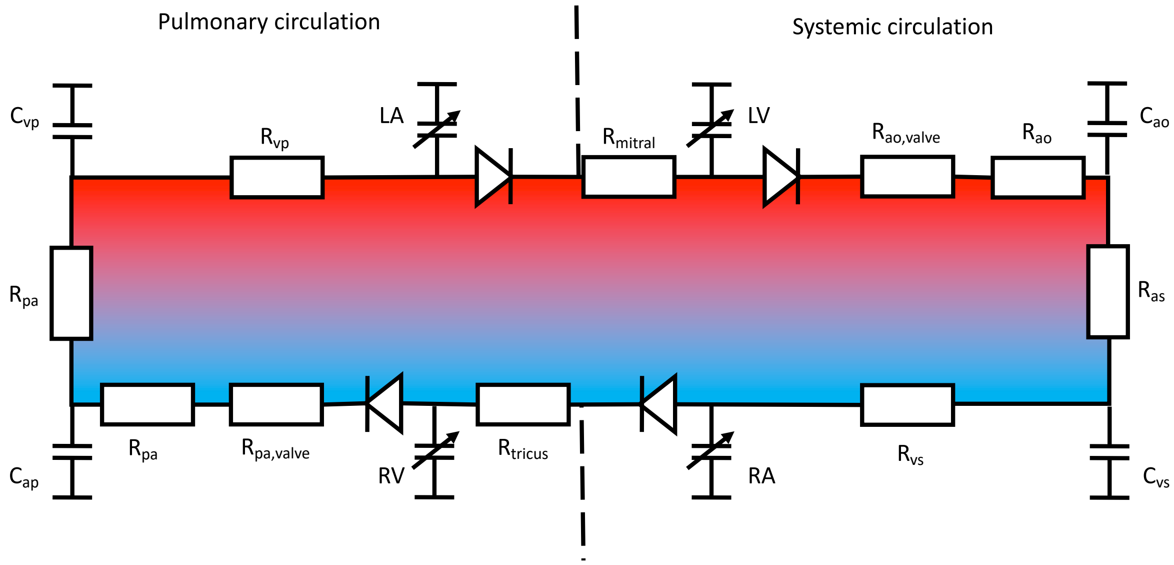


**Figure S2.** Closed-loop model of the circulatory system. Distributed vessel parameters are lumped into discrete resistances and compliances. Parameters given in table S2. Colors represent oxygenated (red) and deoxygenated (blue) blood.

**Table S2.** Parameters of the lumped closed-loop model as shown in fig. S2. $R_{mitral}$: mitral valve resistance, $R_{ao,valve}$: aortic valve resistance, $R_{ao}$: aortic resistance, $C_{ao}$: aortic compliance, $R_{as}$: arterial systemic resistance, $C_{vs}$: venous systemic compliance, $R_{vs}$: venous systemic resistance, $R_{tricus}$: tricuspid valve resistance, $R_{pa,valve}$: pulmonary artery valve resistance, $R_{pa}$: pulmonary artery resistance, $C_{pa}$: pulmonary artery compliance, $R_{ap}$: arterial pulmonary resistance, $C_{vp}$: venous pulmonary compliance, $R_{vp}$, $V_{ao}$, $V_{vs}$, $V_{pa}$ and $V_{vp}$ are unstressed volumes not contributing to the pressure across the corresponding compliance and $V_{total}$ is the total blood volume. Resistances ($R$) in mmHg $\cdot$ s $\cdot$ mL$^{-1}$, compliances ($C$) in mL $\cdot$ mmHg$^{-1}$ and volumes ($V$) in mL

| Parameter | Value |
|---|---|
| $R_{mitral}$ | 0.003 |
| $R_{ao,valve}$ | 0.006 |
| $R_{ao}$ | 0.015 |
| $C_{ao}$ | 2.0 |
| $R_{as}$ | 0.9 |
| $C_{vs}$ | 100.0 |
| $R_{vs}$ | 0.03 |
| $R_{tricus}$ | 0.003 |
| $R_{pa,valve}$ | 0.003 |
| $R_{pa}$ | 0.02 |
| $C_{pa}$ | 10.0 |
| $R_{ap}$ | 0.07 |
| $C_{vp}$ | 15.0 |
| $R_{vp}$ | 0.03 |
| $V_{ao}$ | 800 |
| $V_{vs}$ | 2850 |
| $V_{pa}$ | 150 |
| $V_{vp}$ | 200 |
| $V_{total}$ | 5500 |

## REFERENCES


Balay, S., Abhyankar, S., Adams, M. F., Benson, S., Brown, J., Brune, P., et al. (2021). *PETSc/TAO Users Manual*. Tech. Rep. ANL-21/39 - Revision 3.16, Argonne National Laboratory

Cole, K. S. and Cole, R. H. (1941). Dispersion and Absorption in Dielectrics I. Alternating Current Characteristics. *The Journal of Chemical Physics* 9, 341–351. doi:10.1063/1.1750906

Colli Franzone, P., Pavarino, L., and Taccardi, B. (2005). Simulating patterns of excitation, repolarization and action potential duration with cardiac Bidomain and Monodomain models. *Mathematical Biosciences* 197, 35–66. doi:10.1016/j.mbs.2005.04.003

Fritz, T., Wieners, C., Seemann, G., Steen, H., and Dössel, O. (2014). Simulation of the contraction of the ventricles in a human heart model including atria and pericardium. *Biomech Model Mechanobiol (2014)* 13, 627–641. doi:10.1016/j.pbiomolbio.2015.12.010

Gabriel, S., Lau, R. W., and Gabriel, C. (1996). The dielectric properties of biological tissues: III. Parametric models for the dielectric spectrum of tissues. *Physics in medicine and biology* 41, 2271–2293. doi:10.1088/0031-9155/41/11/003

Gerach, T., Schuler, S., Fröhlich, J., Lindner, L., Kovacheva, E., Moss, R., et al. (2021). Electro-Mechanical Whole-Heart Digital Twins : A Fully Coupled Multi-Physics Approach. *Mathematics* 9, 1–33. doi:10.3390/math9111247

Gurev, V., Pathmanathan, P., Fattebert, J. L., Wen, H. F., Magerlein, J., Gray, R. A., et al. (2015). A high-resolution computational model of the deforming human heart. *Biomechanics and Modeling in Mechanobiology* 14, 829–849. doi:10.1007/s10237-014-0639-8

Keller, D. U. J., Jarrousse, O., Fritz, T., Ley, S., Dössel, O., and Seemann, G. (2011). Impact of physiological ventricular deformation on the morphology of the T-wave: A hybrid, static-dynamic approach. *IEEE Transactions on Biomedical Engineering* 58, 2109–2119. doi:10.1109/TBME.2011.2147785

Keller, D. U. J., Weber, F. M., Seemann, G., and Dössel, O. (2010). Ranking the influence of tissue conductivities on forward-calculated ECGs. *IEEE Transactions on Biomedical Engineering* 57, 1568–1576. doi:10.1109/TBME.2010.2046485

Kerckhoffs, R. C., Neal, M. L., Gu, Q., Bassingthwaighte, J. B., Omens, J. H., and McCulloch, A. D. (2007). Coupling of a 3D finite element model of cardiac ventricular mechanics to lumped systems models of the systemic and pulmonic circulation. *Annals of Biomedical Engineering* 35, 1–18. doi:10.1007/s10439-006-9212-7

Niederer, S. A., Kerfoot, E., Benson, A. P., Bernabeu, M. O., Bernus, O., Bradley, C., et al. (2011). Verification of cardiac tissue electrophysiology simulators using an N-version benchmark. *Philosophical transactions. Series A, Mathematical, physical, and engineering sciences* 369, 4331–4351. doi:10.1098/rsta.2011.0139

Paige, C. C. and Saunders, M. A. (1975). Solution of Sparse Indefinite Systems of Linear Equations. *SIAM Journal on Numerical Analysis* 12, 617–629

Pezzuto, S., Hake, J., and Sundnes, J. (2016). Space-discretization error analysis and stabilization schemes for conduction velocity in cardiac electrophysiology. *International Journal for Numerical Methods in Biomedical Engineering* 32, e02762. doi:10.1002/cnm.2762

Seemann, G., Sachse, F. B., Karl, M., Weiss, D. L., Heuveline, V., and Dössel, O. (2010). Framework for modular, flexible and efficient solving the cardiac bidomain equations using PETSc. In *Progress in Industrial Mathematics at ECMI 2008*, eds. A. D. Fitt, J. Norbury, H. Ockendon, and E. Wilson (Berlin, Heidelberg: Springer Berlin Heidelberg). 363–369. doi:10.1007/978-3-642-12110-4-55

Usyk, T. P., Mazhari, R., and McCulloch, A. D. (2000). Effect of laminar orthotropic myofiber architecture on regional stress and strain in the canine left ventricle. *Journal of Elasticity* 61, 143–164. doi:10.1023/A:1010883920374
